# Appearance Constrained Semi-Automatic Segmentation from DCE-MRI is Reproducible and Feasible for Breast Cancer Radiomics: A Feasibility Study

**DOI:** 10.1038/s41598-018-22980-9

**Published:** 2018-03-19

**Authors:** Harini Veeraraghavan, Brittany Z. Dashevsky, Natsuko Onishi, Meredith Sadinski, Elizabeth Morris, Joseph O. Deasy, Elizabeth J. Sutton

**Affiliations:** 1 Memorial Sloan Kettering Cancer Center, Medical Physics, New York, NY 10065 USA; 2 Present Address: University of Chicago, Radiology, Chicago, USA; 3 Memorial Sloan Kettering Cancer Center, Radiology, New York, NY 10065 USA

## Abstract

We present a segmentation approach that combines GrowCut (GC) with cancer-specific multi-parametric Gaussian Mixture Model (GCGMM) to produce accurate and reproducible segmentations. We evaluated GCGMM using a retrospectively collected 75 invasive ductal carcinoma with ERPR+ HER2− (n = 15), triple negative (TN) (n = 9), and ER-HER2+ (n = 57) cancers with variable presentation (mass and non-mass enhancement) and background parenchymal enhancement (mild and marked). Expert delineated manual contours were used to assess the segmentation performance using Dice coefficient (DSC), mean surface distance (mSD), Hausdorff distance, and volume ratio (VR). GCGMM segmentations were significantly more accurate than GrowCut (GC) and fuzzy c-means clustering (FCM). GCGMM’s segmentations and the texture features computed from those segmentations were the most reproducible compared with manual delineations and other analyzed segmentation methods. Finally, random forest (RF) classifier trained with leave-one-out cross-validation using features extracted from GCGMM segmentation resulted in the best accuracy for ER-HER2+ vs. ERPR+/TN (GCGMM 0.95, expert 0.95, GC 0.90, FCM 0.92) and for ERPR + HER2− vs. TN (GCGMM 0.92, expert 0.91, GC 0.77, FCM 0.83).

## Introduction

Breast cancer is one of the most commonly diagnosed cancers in women and the second most common cause of cancer-related deaths^1^. Although the increasing availability of novel treatment options has helped to improve survival among patients, robust tools are critically needed to effectively monitor treatment response^2^. Miranikova *et al*.^3^ have shown that tumour volumes measured on magnetic resonance imaging (MRI) predict treatment response in neoadjuvant settings. However, accurate and reproducible tumour segmentation is crucial for evaluating breast cancer response to treatments^4^ and to improve surgical outcomes^5^.

Accurate and reasonably fast segmentation is critical for radiomics analysis^6^ which consists of extracting image features from large datasets with the purpose of identifying non-invasive image-based surrogates for diagnosis (differentiating disease aggressiveness) and for predicting treatment response. Radiomics analysis of breast cancers have been used for predicting cancer treatment outcomes^7–9^ and for differentiating between breast cancers by molecular subytpe^10–13^ or for classifying cancers by their aggressiveness^14,15^.

The first and crucial step in extracting the various texture measures is segmentation of the cancer. With the exception of^11,15^, the vast majority of works have employed manual tumour segmentation for radiomics analysis due to the difficultly in ensuring accurate computer segmentations. However, manual delineation is time consuming. Therefore, majority of works^12–14^ including ours^10,16^ have used manual segmentation of one or a few representative slices. Recently, semi-automatic segmentations including GrowCut (GC)^17^ have been reported to produce more reproducible texture features compared with features computed from manually delineated lung tumors^18^, thereby, underscoring the importance and utility of computer-generated segmentations for high-throughput radiomics.

Interactive segmentation methods^19,20^ model the user input to generate more accurate segmentations than fully automatic methods. Thus, the interactive GC method has been shown to produce reasonably accurate segmentations for brain gliomas^17^ and more repeatable segmentations than expert users^21^ for lung cancers. However, as an interactive method adapts its segmentation to user’s inputs, it generates highly variable segmentations, thereby, introducing another source of variability for radiomics and longitudinal analysis of cancers. Previous works, which include^22–25^ have incorporated machine learning to reduce segmentation variability. For example, Veeraraghavan and Miller^23^ developed an active learning-based approach to improve the consistency of segmentation while reducing the number of required user interactions to generate reasonably accurate segmentations of brain cancers. However, repetitive interactions resulting either from the algorithm itself which present as queries or from users can become time consuming particularly for high-throughput radiomics analysis. This in turn limits the applicability of such methods for high-throughput analysis in comparison to fully automatic methods such as unsupervised fuzzy clustering^26^.

We report an approach to improve the accuracy and reproducibility of interactive GC. Specifically, we developed an approach that combines the cancer-specific appearance modeling using multi-parametric Gaussian mixture models (GMM) with GC to constrain the GC segmentation, called GCGMM. Our approach eliminates the need for repetitive user interactions by generating a probabilistic segmentation. The user can select from among multiple segmentations by changing the segmentation probability (or confidence).

The goals of this study were to: (a) develop a reasonably accurate and reproducible approach to generate breast cancer segmentation with variable user inputs, and (b) to assess the feasibility of features extracted from computer-generated segmentation over manual delineation for radiomics-based classification of breast cancers. We compared the results of our approach with the GrowCut (GC) and fuzzy c-means (FCM) clustering^26^. FCM was chosen for benchmarking the performance of GCGMM as the former method has previously been used in radiomics analysis of breast cancers.

## Results

We evaluated the reproducibility of manual delineations produced by multiple users using six consecutive cases with two from ER-HER2+, two from ERPR + HER2− and two from triple negative cancers to benchmark segmentation performance. All raters produced highly variable segmentations. The segmentation concordance measured using the various performance metrics was: Dice overlap coefficient (DSC) (0.78 ± 0.10), mean surface disance (mSD) (1.23 mm ± 0.67 mm), 95% Hausdorff distance (5.04 mm ± 5.9 mm), and volume ratio (VR) (0.16 ± 0.10).

### GCGMM segmentations were significantly more accurate compared with other methods

**Figure 1:**
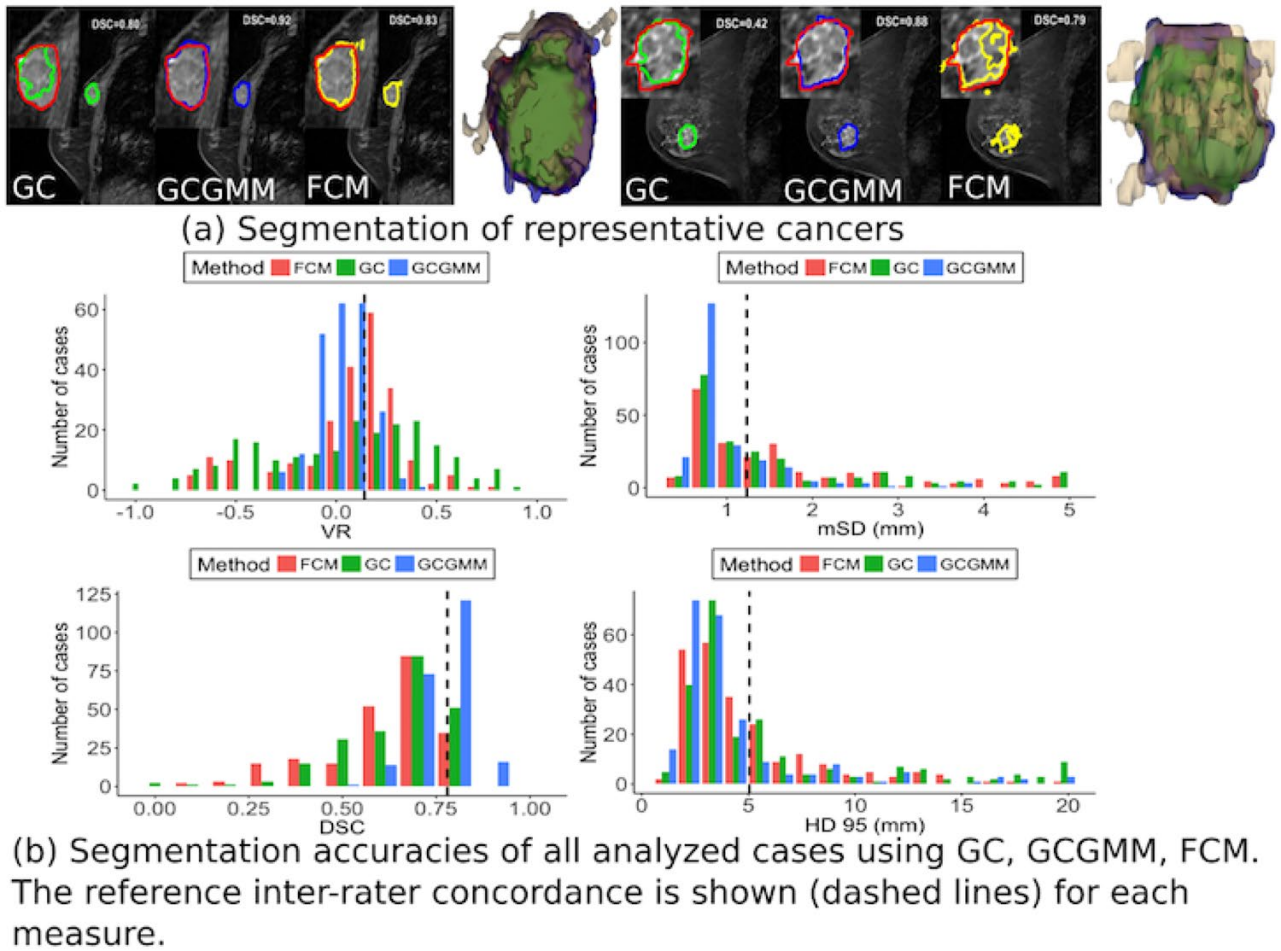
Performance of segmentation methods. (**a**) Example segmentations produced using GrowCut(GC), GC combined with Gaussian mixture models (GCGMM), fuzzy c-means clustering method (FCM) and volumes produced using all methods overlaid with expert delineated volume and (**b**) overall performance of the segmentation methods for all analyzed tumours. The inter-rater segmentation concordance computed using the various metrics is shown for reference using dashed lines.

Figure 1(a) shows segmentations produced using the grow-cut (GC), GCGMM, and FCM methods together with expert delineation for two different tumours. As shown, GCGMM segmentations closely corresponded to the expert delineation while the GC and FCM methods resulted in under- and over-segmentations, respectively. Overall, GCGMM produced significantly higher DSC; significantly smaller mSD, smaller HD95 and lower VR compared with other methods (Fig. 1(b), Table 1).

**Table 1:** Segmentation accuracies generated using GC, GCGMM, and FCM presented using mean and standard deviation (SD).

Analysis	FCM				GC				GCGMM			
	DSC	mSD	HD95	|VR|	DSC	mSD	HD95	|VR|	DSC	mSD	HD95	|VR|
Overall mean	0.66	1.85	5.55	0.27	0.69	2.97	7.38	0.21	0.81***^,^***	1.08**^,^***	4.82**^,^***	0.12***^,^***
SD	0.15	1.31	3.41	0.16	0.15	12.29	14.18	0.18	0.07	0.59	3.67	0.08
Mild BPE mean	0.65	1.89	5.43	0.29	0.70	1.73	8.58	0.20	0.80***^,^***	1.11*^,^***	5.27 *ns*,*ns*	0.13*^,^***
SD	0.15	1.19	3.15	0.16	0.12	1.41	20.98	0.15	0.06	0.62	4.61	0.08
Marked BPE mean	0.68	1.74	5.73	0.25	0.68	3.48	6.41	0.24	0.81***^,^***	1.01*ns*,***	4.44*ns*,*ns*	0.10***^,^***
SD	0.15	1.41	3.88	0.17	0.15	14.84	4.53	0.19	0.07	0.58	2.82	0.07
Mass mean	0.66	1.93	5.63	0.27	0.70	2.34	5.73	0.21	0.82***^,^***	1.02***^,^***	4.24*ns*,*ns*	0.12***^,^***
SD	0.16	1.39	3.66	0.17	0.14	8.49	4.10	0.16	0.07	0.45	2.49	0.08
Non-mass mean	0.68	1.64	5.32	0.27	0.66	4.57	11.64	0.23	0.78*ns*,*	1.24*ns*,*ns*	6.31*ns*,*ns*	0.11***,*ns*
SD	0.14	1.06	2.66	0.15	0.17	18.84	25.63	0.21	0.07	0.84	5.42	0.08
ER-HER2+ mean	0.67	1.77	5.36	0.27	0.69	2.78	7.94	0.22	0.81***^,^***	1.03***^,^***	4.92*ns*,*ns*	0.10***^,^***
SD	0.16	1.32	3.07	0.16	0.14	11.90	16.6	0.17	0.06	0.61	3.88	0.07
TN mean	0.65	2.03	5.15	0.29	0.73	5.41	6.78	0.19	0.82*ns*,*ns*	1.21*ns*,*ns*	4.63*ns*,*ns*	0.14*^,^*
SD	0.14	1.26	2.34	0.16	0.19	20.65	5.85	0.22	0.09	0.52	2.50	0.09
ERPR + HER2− mean	0.65	2.06	6.59	0.29	0.69	2.01	5.54	0.22	0.79*ns*,*ns*	1.18*ns*,*ns*	4.55*ns*,*ns*	0.15*ns*,*ns*
SD	0.14	1.27	4.91	0.17	0.15	2.06	3.72	0.19	0.07	0.52	3.52	0.09

FCM Fuzzy c-means clustering; GC GrowCut; GCGMM GrowCut with Gaussian Mixture Models.

DSC Dice coefficient; mSD mean surface distance; HD95 95^*th*^ percentile of Hausdorff distance; |VR| absolute volume ratio.

Significant differences between GCGMM vs. FCM and GCGMM vs. GC are indicated above each metric for the corresponding analysis after adjusting for multiple comparisons using Bonferroni-Holm correction.

ns *P* ≥ 0.05; *P < 0.05; **P < 0.01; ***P < 0.001.

Only the GCGMM method achieved a better segmentation performance than the inter-rater segmentation concordance using all the performance measures. Furthermore, GCGMM segmentations were more accurate compared with GC and FCM methods for both mild and marked background parenchymal enhancements (Table 1), and for cancers that presented as masses. Finally, GCGMM produced more accurate segmentation of ER-HER2+ cancers compared with both FCM and GC (*P* < 0.001) using all performance metrics.

Fifty one percent of all tumours generated using GCGMM had volumes similar to expert delineation (−0.1< = *VR* < 0.1) with 8% under- (*VR* < −0.1) and 41% over-segmented. In comparison, GC and FCM resulted in 11% and 14% close to expert delineation; 33% and 18% under-segmentations and 56% and 68% over-segmentations, respectively.

### GCGMM produced reproducible segmentations

GCGMM resulted in the most reproducible segmentations (Table 2) using all the performance metrics, including segmented volumes. The precision errors computed using GCGMM segmentations were smaller for all the performance metrics compared with manual delineations. Additionally, FCM that requires minimal user input such as a region of interest (ROI) placed around the tumor still resulted in higher precision errors compared with GCGMM. Similarly, GC, an interactive segmentation method resulted in the largest precision errors shown by both larger %*CV*_*RMS*_ and *SD*_*RMS*_ using all the performance metrics.

**Table 2:** Reproducibility of segmentations generated using multiple raters and by algorithms (GC, FCM, GCGMM) using different user inputs.

Method	*SD*_*RMS*_					%*CV*_*RMS*_				
	DSC	mSD (*mm*)	HD95 (*mm*)	|VR|	Volume (cc)	DSC	mSD	HD95	|VR|	Volume (cc)
Manual	0.084	0.063	4.6	0.10	1.08	11.1	48.3	48.6	62.6	29.4
FCM	0.06	0.91	2.38	0.06	2.46	13.6	31.9	29.7	33.5	36.1
GC	0.10	12.3	13.5	0.14	37.6	19.6	50.0	26.7	64.2	43.8
GCGMM	0.038	0.31	1.33	0.057	1.75	5.07	21.2	20.7	54.3	14.5

*SD*_*RMS*_ Root mean square of standard deviation; %*CV*_*RMS*_ Percentage coefficient of variation in the RMS value for a specific metric FCM Fuzzy c-means clustering; GC GrowCut; GCGMM GrowCut with Gaussian Mixture Models.

DSC Dice coefficient; mSD mean surface distance; HD95 95^*th*^ percentile of Hausdorff distance; |VR| absolute volume ratio.

**Figure 2:**
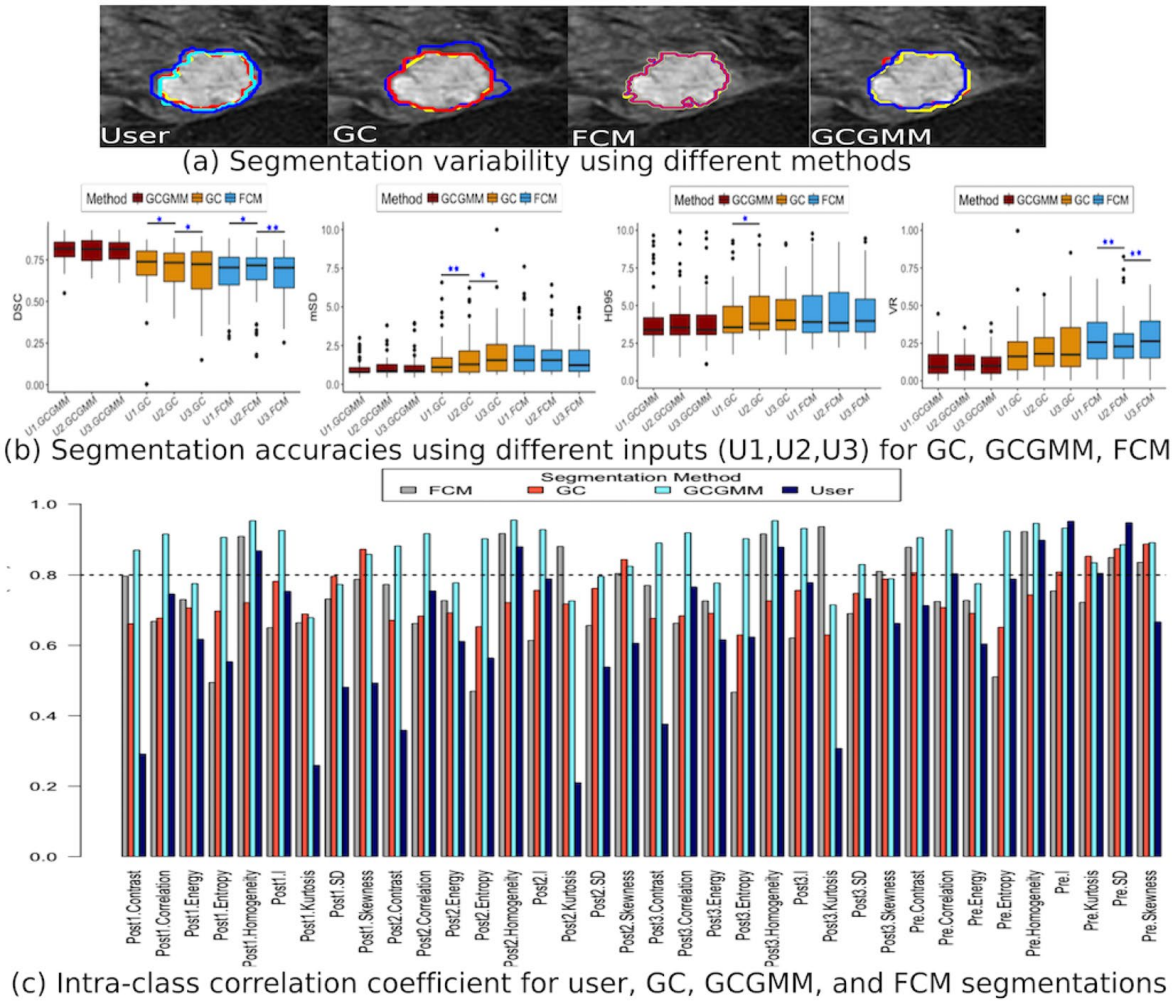
Segmentation variability for the different methods. The inter-rater delineations, and the segmentations generated using three different user inputs are shown in (**a**). The segmentation accuracies achieved by the different methods for the three different user inputs is shown in (**b**) and the segmentations with significantly different accuracies using a given measure are identified, where *P < 0.05 and **P < 0.01. The p-values are reported after adjusting for multiple comparisons using Bonferroni-Holm method. The intra-class correlation coefficient (ICC) of the texture measures computed from the generated segmentations are shown in (**c**).

Figure 2(a) shows the inter-rater segmentation variability for an example case. Computer generated segmentations for GC, FCM, and GCGMM computed using three different user inputs are also shown for comparison. As seen, the GCGMM and FCM segmentations show lower variability compared with either the GC or multi-rater segmentations. As shown in Fig. 2(b), (Table S1), overall, GCGMM achieved more consistent segmentation performance compared with all the analyzed methods.

We measured the reproducibility of the textures extracted from the various segmentations generated using the various methods and with multiple user inputs by computing the intraclass correlation coefficient (ICC) between the texture features. The inter-rater manual segmentations were the least reproducible and achieved the lowest ICC with a median of 0.65 (IQR 0.550.79). The features computed from GCGMM segmentations were the most reproducible with highest ICC with a median of 0.89 (IQR 0.790.925) compared with ICC of features computed from GC median of 0.72 (IQR 0.680.78) and FCM median of 0.73 (IQR 0.660.82). Thirty four out of the 36 features computed using GCGMM method had higher ICC compared with inter-rater manual delineations with the exception of MRI pre-contrast intensity and pre-contrast standard deviation features. Similarly, 33 and 31 features computed using GCGMM had higher ICC compared with FCM and GC method, respectively.

The time required for generating segmentations using GCGMM was 148 secs ± 108 secs compared with FCM (38 secs ± 12 secs) and GC (55 secs ± 25 secs) methods using a HP Z820 PC. Only the GC algorithm was optimized for speed using multi-threading using implementation in C++. The tensor computation was also implemented in C++ for speed. The rest of the algorithm, particularly, Gaussian mixture modeling is implemented in Matlab.

### Classifiers trained using features extracted from computer-generated segmentations were comparable to classifiers trained using features extracted from expert delineations

**Figure 3:**
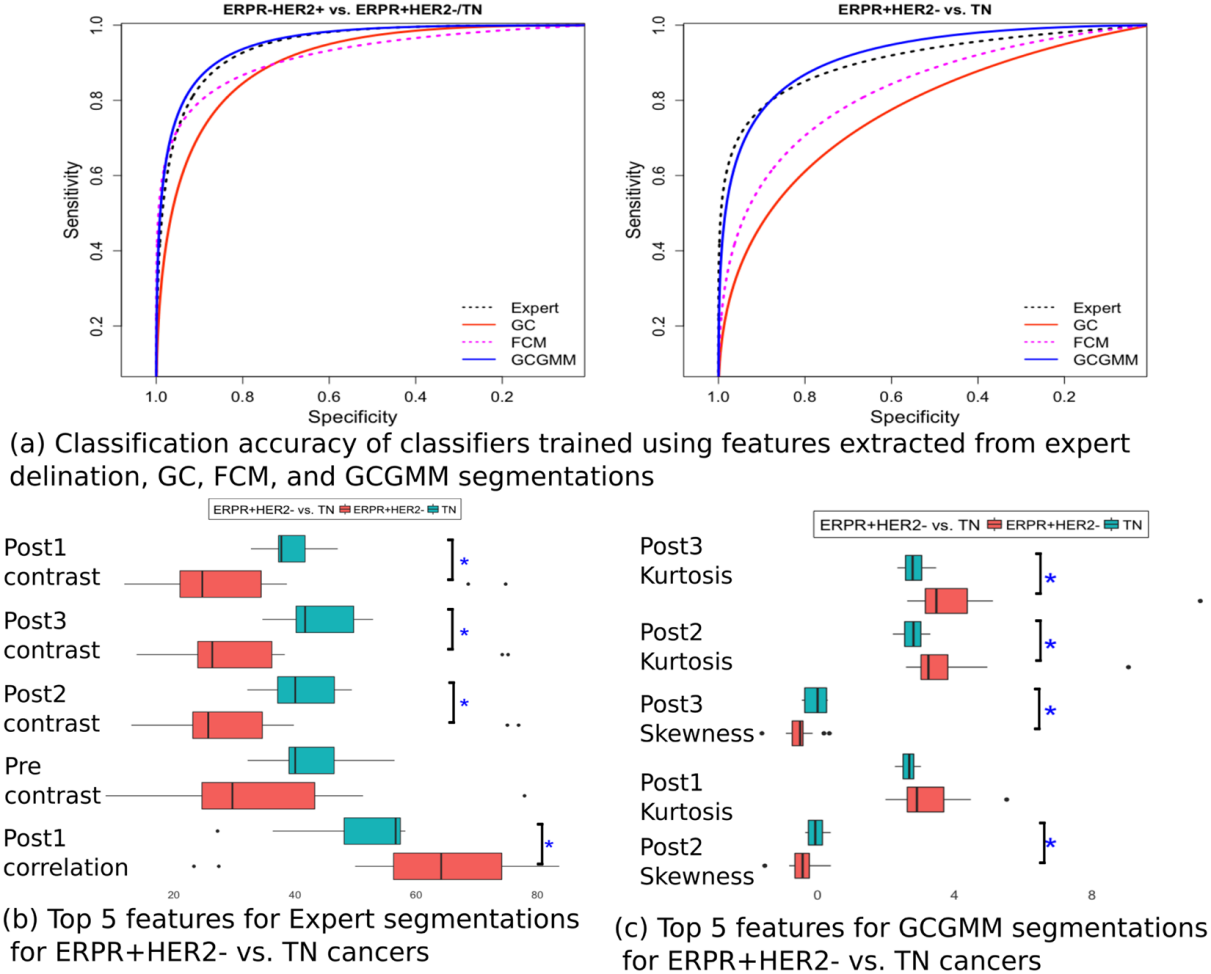
Performance of classifiers trained with textures extracted from different segmentations. (**a**) ROC curves for classifiers trained using features extracted from various segmentations for distinguishing between ER-HER2+ vs. ERPR + HER2−/TN and ERPR + HER2− vs. TN cancers. The five most relevant features and their differences between ERPR + HER2− vs. TN cancers for expert delineated (**b**) and GCGMM segmented tumors (**c**) are also shown.

Classifiers trained using features extracted from GCGMM segmentations achieved the best accuracy for differentiating between the breast cancer molecular subtypes (Table 3, Fig. 3). Furthermore, GCGMM-based classifiers outperformed classifiers that used features computed from expert delineated tumors.

**Table 3:** Classifier accuracies using features computed from different segmentations. TPR - true positive rate, TNR - true negative rate, FPR - false positive rate, FNR - false negative rate, AUC - area under the curve.

Method	ER-HER2+ vs. ERPR + HER2−/TN					ERPR + HER2− vs. TN				
	TPR	TNR	FPR	FNR	AUC (95% CI)	TPR	TNR	FPR	FNR	AUC (95% CI)
Expert	0.85	0.91	0.09	0.15	0.95 (0.91–0.97)	0.78	0.91	0.09	0.22	0.91 (0.79–0.97)
FCM	0.85	0.85	0.15	0.15	0.92 (0.87–0.96)	0.74	0.83	0.17	0.26	0.83 (0.67–0.91)
GC	0.79	0.79	0.21	0.21	0.90 (0.86–0.94)	0.70	0.78	0.22	0.30	0.77 (0.61–0.90)
GCGMM	0.93	0.81	0.19	0.07	0.95 (0.92–0.98)	0.83	0.96	0.04	0.17	0.92 (0.82–0.97)

The ranking of features varied across classifiers (Table 4). Only the features extracted using GCGMM and expert delineation showed significant differences between ERPR + HER2− vs. TN (Table 4). When using the expert delineations, TN cancers had a significantly higher contrast texture compared with ERPR + HER2− cancers (Fig. 3(b)). TN cancers also had a significantly lower first-post contrast MRI correlation (Fig. 3(b)). Four of the top five features computed using GCGMM were significantly different between the two cancers. The TN cancers had significantly lower kurtosis from the second, and third post-contrast MRI, and significantly higher skewness from the second post-contrast MRI (Fig. 3(c).

**Table 4:** Results of Wilcoxon test to assess the difference between ER-HER2+ vs. ERPR + HER2−/TN and ERPR + HER2− vs. TN cancers using top five-most relevant (determined using Gini importance) features extracted using RF classifiers and trained using features generated from the different segmentation methods. P-values are reported after adjusting for multiple comparisons using Bonferroni-Holm method.

Expert	p-Value	FCM	p-Value	GC	p-Value	GCGMM	p-Value
**ER-HER2+ vs. ERPR+/TN**							
Post2 I	0.74	Post3 Kurt	0.56	Post1 Skew	1.00	Post1 I	1.00
Post2 Skew	0.31	Pre Kurt	0.36	Pre Kurt	1.00	Post3 I	1.00
Post1 I	1.00	Post2 Kurt	0.56	Pre Contrast	1.00	Post2 I	1.00
Post1 Corr	1.00	Post1 Kurt	0.56	Post1 Kurt	1.00	Pre Energy	0.19
Post1 Entropy	1.00	Post3 SD	0.56	Post2 Skew	1.00	Post3 Skew	1.00
**ERPR + HER2− vs. TN**							
Expert	p-Value	FCM	p-Value	GC	p-Value	GCGMM	p-Value
Post1 Contrast	0.04	Post3 Kurt	0.27	Post3 Homogeneity	0.71	Post3 Kurt	0.01
Post3 Contrast	0.02	Post3 SD	0.65	Post3 Skew	0.71	Post2 Kurt	0.01
Post2 Contrast	0.04	Post2 Skew	0.65	Post2 Skew	0.58	Post3 Skew	0.01
Pre Contrast	0.08	Post1 Kurt	0.32	Pre SD	1.00	Post1 Kurt	0.16
Post1 Corr	0.04	Post1 Skew	0.65	Post2 I	1.00	Post2 Skew	0.01

FCM: Fuzzy c-means; GC: Grow-Cut; GCGMM: Grow-Cut with Gaussian Mixture Models

Pre: Pre constrast MRI; Post1: first post-contrast MRI; Post2: second post-contrast MRI; Post3: third post-contrast MRI

I: intensity; skew: skewness; corr: correlation; kurt: kurtosis; SD: standard deviation.

## Discussion

We developed an appearance constrained interactive segmentation method, which generated accurate for breast cancers with three different molecular subtypes as well as with different tumour presentations (mass and non-mass) and background parenchymal enhancement (mild and marked). GCGMM produced reproducible segmentations with least precision errors compared to manual, FCM, and GC segmentation methods. Our method was significantly more accurate than GC^20^ and FCM^26^ both of which have been used in various radiomics applications including the lung^21^ and breast cancers^11,15^.

GCGMM resulted in lowest %*CV*_*RMS*_ and lowest *SD*_*RMS*_ using all performance metrics compared with other segmentation methods. The volume precision errors using GCGMM were the lowest (%*CV*_*RMS*_ = 14.5%) compared with all methods including inter-rater segmentations. Similarly, the Hausdorff distance errors were also the lowest with (%*CV*_*RMS*_ = 20.7%) using GCGMM compared with (%*CV*_*RMS*_ = 48.6%) when using manual delineations. The precision errors computed using the GC method were high and more comparable to the inter-rater delineations than the FCM or GCGMM methods, clearly underscoring the fact that an interactive method such as GC is impacted by variability in user inputs. Finally, texture measures computed from GCGMM were more reproducible compared with GC and FCM segmentations as well as inter-rater delineations and resulted in the highest ICC. Ultimately, features computed using the GCGMM segmentations produced the best classification accuracy in a radiomics classification task involving cancer molecular subtypes and only the features computed using GCGMM besides the expert delineation were able to capture significant differences between the studied breast cancer molecular subtypes. Our results demonstrate that GCGMM is a feasible method for generating accurate and reproducible segmentations for breast cancer radiomics analysis. GCGMM method took longer to compute compared with the GC or the FCM method. However, the computation time on average was under 3 mins. We did not perform any code optimization while computing the run times.

Our method resulted in fewer over- or under-segmentations compared with either GC or FCM. We developed an in-house GUI for interactive selection of the appropriate volumetric lesion segmentation, which enables simultaneous radiologist validation. Given the evidence of the importance of tumour volumes in assessing treatment response in neoadjuvant chemotherapy^3^ and for improving surgical outcomes^5^, an approach such as ours can potentially benefit the translation of computer-aided techniques into clinical settings. We are currently evaluating our approach among a different cohort of breast cancer patients imaged prior to and following treatment with neoadjuvant chemotherapy.

Repeated interactions as needed in GC^20^ can be especially cumbersome when segmenting large datasets. Fully automatic methods^3,8,9,26,27^ need little to no user interaction but may lead to less accurate results as they fail to match the expert’s assessment of tumour boundary. In this report, we improved the performance, in both accuracy and reproducibility of an interactive method while limiting user input (brush strokes or rectangular ROI enclosing the tumour) by using a simple cancer-specific appearance modeling approach in favor of voxel-wise shallow learning^28–30^ and more recent deep learning methods^31–33^. Our approach takes advantage of the temporal variability in the lesion appearance and derived image representations such as the temporal difference^13^ and tensor-derived scalar images inspired by^34,35^ that seek to differentiate the tumour’s appearance from its background. Our results show that our approach generates consistently accurate segmentations for a variety of tumour molecular subtypes, patterns of enhancement, and BPE. Prior works on breast cancer segmentation typically focused on specific tumour types such as ER(+), node negative tumours as in^28^ or tumours with specific appearance including mass and non-mass enhancing patterns as in^30^, datasets with malignant and benign breast cancers^34,36^.

Prior works including^18,21^ showed that GC segmentations were more repeatable than manual delineations produced by different users both in terms of segmentation variability and texture feature reproducibility. Our work went a step further to improve the reproducibility of GC using GCGMM and assessed the performance difference in a radiomics task when using features computed from the different segmentations. Our results show that features computed from any of the analyzed algorithmic methods produced similar results as manual delineations and can in fact yield better results, as in the case of GCGMM. Furthermore, our work illustrates the utility of using volumetric measurements for improving classification accuracy. Previously, we used a different cohort of patients^10^ to differentiate between the breast cancer subtypes and our results clearly demonstrate the performance improvement.

Four out of five top ranked features extracted using GCGMM and expert delineation were significantly different between ERPR+ and TN cancers. Similar to the findings from^11,12^ which found TN cancers to be more heterogeneous, our results show that using both expert delineated and GCGMM segmentations, TN cancers were associated with higher heterogeneity, namely, larger contrast and lower kurtosis. Finally, it is interesting to note that classifiers trained using different segmentations resulted in different ranking of features.

Our work has the following limitations. First, the dataset was imbalanced between the different molecular subtypes which required data balancing using the SMOTE technique^43^. Second, experts generated delineations in consensus which prevented us from studying the variability of auto-generated segmentation with respect to inter-rater variability. We tried to address this issue by benchmarking the inter-rater variability using a small number of randomly chosen cases. Nevertheless, we evaluated our approach on a reasonably diverse set of tumours and performed a systematic evaluation starting from auto-generated segmentation to assessing feasibility of features extracted from such segmentations in a radiomics task.

## Methods

### Study design and patients

Our institutional review board approved our HIPAA-compliant retrospective study. A retrospective cohort of 75 patients diagnosed with pathologically-proven invasive ductal breast carcinoma between 2006–2011 were analysed. Tumour subtypes were identified through immunohistochemistry with known ER, PR, and HER2/neu receptor status. Inclusion criteria were: (i) preoperative bilateral breast MRI, (ii) no prior history of cancer, (iii) no known BRCA mutation, and (iv) no current use of hormonal therapy. Our study population consisted of 56 HER2 receptor positive (HER2+, n = 56), 15 estrogen positive (ER) and progesterone receptor (PR) positive, and 9 triple negative (TN, n = 9) tumours. Thirty-six patients used in this study overlapped with those used in^10^ and all the 15 ERPR+ patients overlapped with those used in^16^.

Sagittal T1-weighted, fat-suppressed 2D multi-slice (40–50 slices) images were acquired with a 1.5-T MRI system (Signa or Signa HDX; GE Medical Systems) using a dedicated 8-channel surface breast coil before and continuously at three times after the intravenous administration of 0.1 mmol gadopentetate-dimeglumine per kilogram body weight (Magnevist) using the following scan parameters: repetition time (ms)/echo time (ms), 7.4/4.2; flip angle, 10°; bandwidth, 32 kHz; field of view 18–22 *cm*; acquisition matrix 256 × 192; slice thickness, 3 mm; temporal resolution 90 s.

A radiologist (EJS) with six years of experience reading breast MRIs who was blinded to cancer molecular subtype classified all tumors as having mass or non-mass enhancement (NME). BPE was also assessed as mild or marked BPE. Tumours classified by the radiologist as having both mass and non-mass enhancement were classified as NME for the purpose of analysis. Two radiologists (EJS, BZD) generated volumetric manual delineation of the tumours using the first post-contrast T1w MRI in consensus using ITK-SNAP^37^ software which served as the ground truth segmentation.

### User inputs for segmentations

**Figure 4:**
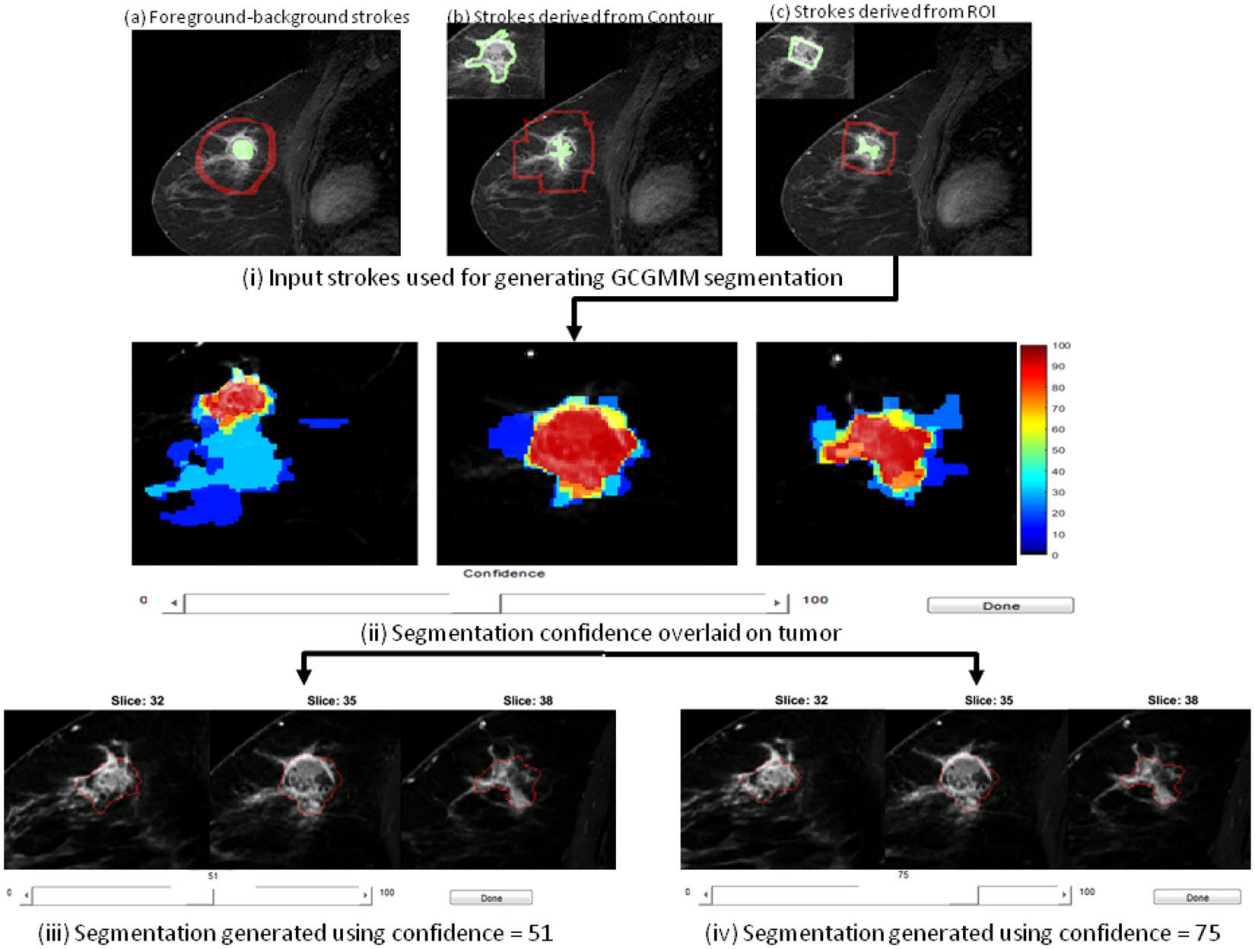
Workflow diagram. (i) Inputs used for generating segmentations, (ii) confidence map computed from GCGMM using region of interest refined input from (i) c, and segmentations generated using two different confidence thresholds (iii,iv) for a triple negative breast cancer.

The goal of the user input experiment was to study the robustness of the algorithms in generating volumetric segmentations with varying user inputs. Therefore, we used the following strategy to evaluate the segmentation performance. Three users (two radiologists and computer scientist) produced inputs for the segmentation method. User EJS traced a contour delineating the tumor on a single slice. The second user input was placed to roughly enclose the tumor. The main difference between the first and second input was that while the first user carefully followed the tumor boundary including spiculations, the second input was a rough polygonal region of interest (ROI) that did not follow the exact tumor boundary and simply enclosed the tumor. The third input (tumor/background) consisted of a contour drawn within the tumor. Additionally, the third user placed a background contour outside the tumor. The users’ inputs are shown in (Fig. 4(i)).

GC^20^ employs competitive region growing starting from user interactions to produce segmentations according to user preference. Our implementation available in 3DSlicer^17^ for scalar images can use multiple rounds of user inputs to produce a final segmentation. We restricted the user inputs to be presented once during initialization to a single representative slice to make the inputs as close to a fully automatic method as possible. Furthermore, we implemented an automatic background stroke extraction to limit user effort to providing only a rectangular ROI enclosing the tumour.

Our method automatically converted the ROI and contour inputs to extract foreground and background strokes as follows. Foreground strokes were computed from the user contour by extracting the morphological skeleton using *r* − 1 iterations, where *r* corresponds to the half of maximum equivalent contour diameter. The background labels were extracted by subtracting two sets of automatically extracted ROIs computed by dilating the original user-drawn ROI (or contour) using (*d*_1_ = *r*) and (*d*_2_ = *max*(2, *r* − 2)) iterations. The user input enclosing the tumor for the contour and ROI inputs were subjected to one iteration of morphological erosion to ensure that the extracted foreground strokes were contained within the tumour. Next, the foreground strokes were drawn as perpendicular lines extending from the centroid and till the minor axis length of the eroded ROI. The three inputs for an example case are shown in Fig. 4(i).

The inputs for the FCM method consisted of a rectangular ROI extracted by computing the bounding box enclosing the background strokes.

### Segmentation Method

Eight feature images consisting of pre, and three post-contrast MRI, three temporal difference images (computed per voxel as, *ε*_*t*_ = (*I*_*t*_(*x*) − *I*_0_(*x*))^2^, where, *I*_*t*_ was the post-contrast image at time *t* and *x* the voxel location), and a trace image computed from tensor representation of the DCE-MRI were used in the analysis. A voxel-wise tensor was computed from a voxel-wise covariance matrix

$$A=[\begin{array}{cccc}{i}_{0}^{2} & {({i}_{0}-{i}_{t})}^{2} & \ldots  & {({i}_{0}-{i}_{k})}^{2}\\ \vdots  & \ldots  &  & \vdots \\ {({i}_{k}-{i}_{0})}^{2} & \ldots  &  & {i}_{k}^{2}\end{array}],$$

where, *t*_*i*_ was the intensity of a voxel at time *t*. Eigen decomposition of *A* using the top three eigenvalues produced the temporal tensor at each voxel from which the trace image was computed. The trace image summarized the variation in the contrast uptake within the tumour and in the normal parenchyma.

All eight feature images were used for producing segmentation using GC, FCM, and GCGMM methods. FCM clustering used the same parameter settings as used in^26^.

The GCGMM method produced tumour segmentation through a weighted combination of GC segmentations from individual feature images with GMM-based voxelwise classification using:

1 $$L=\frac{1}{N}\sum _{i=1}^{N}{S}_{i}\times (1-\gamma )+G\times \gamma  > \omega ,$$

where, *S*_*i*_ is the GC segmentation for feature image *i*, *G* the GMM model-based segmentation, *N* the number of feature images, and *ω* = 0.6 is an empirically chosen default confidence threshold. The parameter *γ* weights the contribution of GMM and GC segmentation. It corresponds to the *F*_*β*=0.5_ measure^38^ that emphasizes precision over recall to account for large data imbalance between cancer and normal voxels. One GMM model is trained per tumor where the GMM model contains all the features as a vector. Therefore, the *γ* values were chosen per tumor. In general, the *γ* values ranged between 0.09 to 0.75 with mean value of 0.37 ± 0.16 for all the analyzed cases.

The final segmentation was produced by the weighted sum of GC segmentations for each feature image with the GMM-based voxel-wise classification. An alternative approach would be to produce a single GC segmentation by using all the feature images simultaneously (with equal weights) and combining that with the GMM-based classification. We chose the former approach as we hypothesized that the latter approach where all features are weighted equally would result in an under-segmentation as only voxels that are highly similar to the user-labeled tumor voxels and with largest feature distances from background voxels would be labeled as tumour.

We developed a graphical user interface in Matlab (Fig. 4(iii,iv)) that allows a user to dynamically change the confidence threshold *ω* and produce the desired segmentation.

#### Multi-Parametric Gaussian Mixtures Model-based Tumour Extraction

Multi-parametric Gaussian Mixture Models (GMM) were extracted from the feature images using tumour and background input labels. The GMM model parameters, namely, the mean (*μ*), covariance (Σ), mixing weights (*w*), and the number of components (*n*), were automatically extracted from the data. Akaike Information Criterion (AIC) was used to select the appropriate number of mixture components for each GMM from (n = 2, 3, 4). Three was the most frequently selected number of components for tumour and background. GMM models for the tumour and background were computed using expectation maximization (EM) algorithm. The extracted GMM model was then used to produce voxel-wise labelling throughout the entire image. A voxel *x* was assigned tumour or background label to produce a GMM label image *G* using,

2 $$G(x)=(\begin{array}{ll}{\rm{if}}\,{\rm{k}}\,({\rm{x}},{\rm{T}}) > {\rm{k}}({\rm{x}},{\rm{B}}), & {tumour}\\ \mathrm{otherwise}, & {background},\end{array}$$

where $k(x,T),k(x,B)$ are the similarity distances of a voxel *x* computed with respect to the tumour *T* and the background *B* models. To limit the number of false positives, we required that the tumour probability *k*(*x*, *T*) > *τ*, where *τ* = 0.75.

#### Metrics for evaluating segmentation accuracy

Algorithm generated segmentations *A* were compared with radiologist delineated segmentation *G* using spatial overlap computed using the Dice coefficient $(DC=\frac{2\ast A\cap G}{(A\cap G+A\cup G)})$, a volume-based measure called the absolute volume difference ratio $(|VR|=\frac{|v(A)-v(G)|}{0.5\ast (v(A)+v(G))})$ and two distance measures namely, mean surface distance (mSD) and the 95% Hausdorff distance (HD95). HD95 was defined as 95^*th*^ percentile distance over all point distances in contour *X* to its closest point in contour *Y*:

3 $$HD95=\mathrm{95 \% }(\mathop{{\rm{\min }}}\limits_{y\in Y}\,d(x,y))\forall x\in X,$$

where *d*(*x*, *y*) is the distance between the points *x* and *y* in *X* and *Y*, respectively. The mean surface distance between two contours *X* and *Y* is defined as:

4 $$mSD(X,Y)=\frac{1}{|X|}\sum _{x\in |X|}\mathop{{\rm{\min }}}\limits_{y\in Y}\,d(x,y)$$

Large values of the Dice and small values of mSD, HD95, and |VR| indicate high accuracies. The 95^*th*^ percentile Hausdorff distance was used as this is more robust to outliers as explained in^39^.

#### Metrics for evaluating segmentation reproducibility

Segmentation reproducibility resulting from the various methods using multiple user inputs was measured by computing the root mean square (RMS) of the coefficient of variation (%*CV*_*RMS*_) and the RMS of standard deviation (*SD*_*RMS*_) in the segmentation metrics and as described in^40,41^. We used the %*CV*_*RMS*_ as this measure has been shown to be a conservative measure of segmentation reproducibility in^41^. CV is a measure of relative variability and is defined as the ratio of the standard deviation to the mean. The %CV measures for each method *i* and patient *p* using a segmentation metric ${M}^{j}=\{j=DSC,mSD,HD\mathrm{95,}|VR|\}$ were computed as,

5 $$ \% C{V}_{p}({M}^{j})=\frac{\overline{{M}_{p}^{j}}}{\mathop{{M}_{p}^{j}}\limits^{\sim }}\times \mathrm{100,}$$

where, $\overline{{M}_{p}^{j}}$ is the standard deviation in the metric *M*^*j*^ for the multiple user input trials in a given patient *p*, and $\mathop{{M}_{p}^{j}}\limits^{\sim }$ is the mean value of that metric for those same trials and patient. The RMS value for the %CV for each segmentation metric was then computed as,

6 $$ \% C{V}_{RMS}({M}^{j})=\sqrt{\frac{1}{N}\sum _{p\mathrm{=1}}^{N}( \% C{V}_{p}{({M}^{j})}^{2})}\mathrm{.}$$

The RMS SD for each segmentation metric was computed as,

7 $$RM{S}_{SD}=\sqrt{\frac{1}{N}\sum _{p\mathrm{=1}}^{N}{\overline{{M}_{p}^{j}}}^{2}}\mathrm{.}$$

### Radiomics feature extraction and classification

Thirty-six texture features were computed from the DCE-MRI consisting of four first order textures (mean, standard deviation, kurtosis, and skewness) and five second order Haralick texture measures (energy, entropy, correlation, homogeneity, and contrast) from each MR image sequence. The Haralick textures were computed from a gray-level co-occurrence matrix after rescaling the images (0–255) and using 24 histogram bins. Texture measures were computed within the volumetrically segmented tumours using manual, FCM, GC, and GCGMM methods for all the trials resulting in 27000(36 × 3 × 3 × 75 + 36 × 75) texture values. Reliability of the computed textures resulting from segmentations generated by using multiple user inputs was measured by computing the intra-class correlation coefficient (ICC) as used in previous studies^18^.

Random forest classifiers^42^ (with 100 trees and default parameters) were computed using texture measures extracted using each segmentation generated from stroke inputs for distinguishing between (a) HER2+ vs. ERPR+/TN, and (b) ERPR+ vs. TN. Datasets were balanced using the synthetic minority oversampling technique (SMOTE)^43^. Classifier accuracy was evaluated using leave-one-out cross-validation (LOOCV).

### Statistics

Associations between categorical measures (segmentation method, user input trial, molecular subtype, enhancement) and continuous variables (DSC, mSD, and VR) were studied using Kruskal-Wallis tests. Paired associations between continuous variables were analyzed using Wilcoxon rank sum test. P values of <0.05 were considered to be statistically significant. Bonferroni-Holm correction was applied to account for multiple comparisons. All statistical analysis was computed using R statistical software^44^.

### Data availability statement

All of the generated segmentation metrics and texture measures are available in supplementary data. The R code used for performing the statistical analysis is available from the github repository https://github.com/harveerar/SciRepStatAnal/.

### Conclusions

We developed a cancer-specific appearance constrained interactive segmentation method for generating volumetric delineations of breast cancers from DCE-MRI. We performed a systematic evaluation of the method starting from segmentation performance, the influence of multiple user inputs on segmentation differences, and its utility for a radiomics task. Our results show that the GCGMM segmentations were accurate, reproducible and a classifier trained using features extracted from those segmentations were as good or better than classifier trained using features extracted from expert delineations for differentiating between breast cancer molecular subtypes.

## Electronic supplementary material

SI Data 1

SI Data 2

SI Data3

SI Data4

SI Data5

SI Data6

Supplementary Materials
