# Supplementary material for: Appearance Constrained Semi-Automatic Segmentation from DCE-MRI is Reproducible and Feasible for Breast Cancer Radiomics: A Feasibility Study
Source: Sci Rep. 2018 Mar 19;8:4838. doi: 10.1038/s41598-018-22980-9 (PMC5859113; doi:10.1038/s41598-018-22980-9)
Supplement: Supplementary file 7 — Supplementary Materials [file 41598_2018_22980_MOESM7_ESM.docx]

**Appearance constrained semi-automatic segmentation from DCE-MRI is reproducible and feasible for breast cancer radiomics: a feasibility study**

Harini Veeraraghavan, Brittany Z. Dashevsky, Natsuko Onishi, Meredith Sadinski, Elizabeth Morris, Joseph O. Deasy, Elizabeth J. Sutton

Supplementary Materials

Supplementary Tables:

S1 Table

P values computed using Wilcoxon rank sum tests comparing the segmentation accuracies of GC, GCGMM and FCM methods when using different user inputs. P values were adjusted for multiple comparisons using Bonferroni-Holm method. DSC: Dice overlap coefficient, mSD: mean surface distance, HD95: 95% Hausdorff distance, VR: volume ratio

| Input | FCM | | | | GC | | | | | | GCGMM | | | |
| --- | --- | --- | --- | --- | --- | --- | --- | --- | --- | --- | --- | --- | --- | --- |
|  | DSC | mSD | HD95 | \|VR\| | DSC | mSD | HD95 | | | \|VR\| | DSC | mSD | HD95 | \|VR\| |
| Contour vs. ROI | 0.07 | 0.09 | 0.5 | 0.22 | 0.01 | 0.004 | 0.006 | | 0.37 | | 0.25 | 0.06 | 0.08 | 0.81 |
| ROI vs. Tumor/background | 0.01 | 0.83 | 0.4 | 0.001 | 0.04 | 0.02 | 0.14 | 0.44 | | | 0.54 | 0.48 | 0.60 | 0.81 |
| Contour vs. Tumor/background | 0.002 | 0.63 | 0.5 | 0.007 | 0.28 | 0.10 | 0.07 | 0.44 | | | 0.52 | 0.16 | 0.08 | 0.43 |

Supplementary datasets:

SI Data1: Segmentation accuracies computed against expert delineation for all analyzed computer-based segmentation methods generated using multiple user inputs are in SIData1.xlsx

SI Data2: Texture data computed for the segmented tumors using all analyzed computer-based segmentation methods for various user inputs is in SIData2.xlsx

SI Data3: Texture data for ERPR-HER2+ vs. ERPR+HER2-/TN cancers augmented with 150% using SMOTE method for all analyzed computer-based segmentation methods is in SIData3.xlsx

SI Data4: Texture data for ERPR-HER2+ vs. ERPR+HER2-/TN cancers without data augmentation using all analyzed computer-based segmentation methods is in SIData4.xlsx

SI Data5: Texture data for ERPR+HER2- vs. TN cancers augmented with 150% using SMOTE method for all analyzed computer-based segmentation methods is in SIData5.xlsx

SI Data6: Texture data for ERPR+HER2- vs. TN cancers without data augmentation for all analyzed computer-based segmentation methods is in SIData6.xlsx
